# Characteristics and determinants of community physiotherapy utilization and supply

**DOI:** 10.1186/s12913-019-3994-4

**Published:** 2019-03-14

**Authors:** Chidozie Mbada, Abraham Olawuyi, Olufemi O. Oyewole, Adesola C. Odole, Abiola O. Ogundele, Francis Fatoye

**Affiliations:** 1 Department of Medical Rehabilitation, Obafemi Awolowo University, Ile-Ife, Nigeria; 2 Department of Physiotherapy, Olabisi Onabanjo University Teaching Hospital, Sagamu, PMB 2001 Nigeria; 3 Department of Physiotherapy, University of Ibadan, Ibadan, Nigeria; 4 Department of Health Professions, Manchester Metropolitan University, Manchester, UK

**Keywords:** Demand and supply, Physiotherapy services, Community physiotherapy, Rural community, Nigeria

## Abstract

**Background:**

Demand for Physiotherapy is on the rise due to increasing ageing population and consequent disability and morbidity. However**,** the costs of healthcare in developing countries are rising, and healthcare resources are limited making the supply of Physiotherapy services challenging in rural communities. Availability of Physiotherapy may help to reduce the burden of disability and enhance efficiency of healthcare systems. This study investigated the characteristics and associations of utilization and supply of community Physiotherapy in Nigeria.

**Methods:**

Cross-sectional survey of 336 consenting community dwelling individuals from three selected communities in Nigeria was carried out**.** A three-section validated self-developed questionnaire which sought information on socio-demographics, utilization and supply of community Physiotherapy, as well as how to improve community Physiotherapy services was used. A household was used as the primary sampling unit in the study. Inferential and Descriptive statistics were used to assess the data.

**Results:**

Lifetime, 12-month and point utilization of physiotherapy was 21.7, 7.4 and 2.7% respectively. Physiotherapy utilization was significantly associated with level of education (*p* = 0.007), belief on pain as “spiritual” (*p* = 0.020) and religious belief (*p* = 0.001). The respondents with primary, secondary and tertiary education were 14.3, 13.9 and 26 times more likely to utilize physiotherapy services, respectively. Those who ‘agree’ or were ‘not sure’ that their religious belief was against physiotherapy were 92 and 83% less likely to utilize physiotherapy services, respectively compared with those who ‘disagree’. Availability and supply of Physiotherapy services were mostly at the township teaching hospital (47.9%) and private hospitals (20.5%). The supply of Physiotherapy services within the communities was mostly on temporary basis (24.7%) and through visiting Physiotherapists (21.4%). Physiotherapy services utilized was mainly exercise (46.6%) and soft tissue mobilization (41.1%). Travel costs (32.6%), time constraints (27.9%) and work commitments (24.8%) were the constraints for Physiotherapy utilization while positive beliefs and higher education improved Physiotherapy utilization.

**Conclusions:**

Utilization and supply of Physiotherapy services in Nigerian rural community was low. Low utilization of Physiotherapy services in Nigerian rural communities were most significantly influenced by low educational status and beliefs about pain.

**Electronic supplementary material:**

The online version of this article (10.1186/s12913-019-3994-4) contains supplementary material, which is available to authorized users.

## Background

Equitable access to health which is defined as “the capacity of, and opportunity for all individuals to access health care services of similar quality, regardless of barriers” [1] has been advocated for by World Health Organization (WHO). Health systems around the world are looking to improve access to health services and health system effectiveness [2]. Unfortunately, anecdotal evidence suggests that Nigeria is yet to achieve universal healthcare coverage especially in the rural communities. Most Nigerian communities are rural and are characterized by Spartan living standards, as well as, ageing population with disabilities [3, 4]. Living with disability among rural dwellers is a key factor that might lead to upsurge in utilization of healthcare including Physiotherapy amongst the population, in consonance with reports on increasing demand for Physiotherapy services globally [5, 6]. Literature asserts that the upsurge in utilization of Physiotherapy is mostly associated with the increasing ageing population, population growth, rising incidences of chronic disease and survival of accidents or illness [7, 8]. Since young adults are also exposed to incidence of chronic diseases and accidents, their demand for or utilization of physiotherapy is not well documented. Young people are faced with greater risk of morbidity and mortality associated with violence, mental health, and reproductive health problems which may require physiotherapy [9]. Therefore, information on utilization, access to, or factors restraining physiotherapy provision among this age demographic are needed to facilitate resource allocation for effective physiotherapy services.

The demand-supply disequilibrium for Physiotherapy services represents a momentous shortcoming in sub-Saharan Africa [10]. Although, the number of practicing Physiotherapists have increased in some countries [11, 12], the workforce growth is unlikely to overcome existing or future shortages [13]. This is because Physiotherapy services (or supply) have not kept pace with the increasing demand, causing an access (right to use) challenge in most settings [6, 10, 14]. Generally, determinants of utilization and supply of Physiotherapy services are multifarious and widely varied in different countries [15]. Some of these factors are aging workforce and attrition [13], lack or poor remuneration and recognition, workforce shortages, dearth of locum opportunities and rural-urban related factors [14], cost, waiting time, location [16], travel burden, flexibility in work hours, professional support and development, as well as autonomy of practice [17]. Other factors include insurmountable workloads, limited access to continuous professional development and narrow opportunity for career progression, non-availability of employment openings for partners, perceived scarcity of quality secondary schools, and intention to travel [18, 19]. Additionally, context-specific factors contributing to demand-supply shortfall of Physiotherapy services in Nigeria include lack or absence of Physiotherapy services, inadequate knowledge of scope and role of Physiotherapy, incongruous health seeking behaviour and stigmatization, and poor referral [20–22]. Anecdotally, the use of mobility aids such as walkers, canes, crutches and other assistive devices in the Nigeria’s context are viewed in negative light, as users are viewed and related to, as significantly disabled, similar to accounts reported among rural and minority populations in other studies [23, 24].

Limited availability or nonexistence of Physiotherapy services seems to be more apparent in rural than urban settings in Nigeria, as most Physiotherapy facilities are located in urban cities, similar to reports from developing countries [4, 20, 25]. Workforce misdistribution and lack of incentivitization and required skills to practice remain as challenges to having equitable Physiotherapy services in rural population, especially, in the resource-constrained countries [4, 10, 20]. The Australian Physiotherapy Association [26] reported that physiotherapists employers in rural and remote areas often struggled to recruit and retain physiotherapists. In order for physiotherapists to practice in these areas, they require unique skills which can only be attained through experience in the rural setting [26].

Despite available information and anecdotes that residents of rural communities exhibit higher health needs and experience poorer health outcomes [20, 25], many of these communities do not have access to a wide range of health services compared with the urban communities [27–29]. In comparison with other health care professions, provision of Physiotherapy services in rural communities is severely in short supply [30]. Unfortunately, literature is sparse on determinants of utilization and supply of rural Physiotherapy services, especially in the context of low-and-middle-income countries such as Nigeria. Empirical analysis of utilization and supply of Physiotherapy services in rural settings may inform the development and implementation of effective and efficient health policy to improve the health outcomes of individuals in such communities. The objective of this study was to investigate the characteristics and associations of utilization and supply of community Physiotherapy in Nigeria.

## Methods

A door-to-door cross-sectional survey was conducted among rural community dwelling individuals from three communities (Edunabon, Ipetumodu and Moro) in Ife-North Local Government Area (LGA), Osun State in Southwestern, Nigeria. These communities were purposively selected for meeting certain features that are characteristic of rural settings in the Nigeria’s context, such as having small population densities and settlement size, lack of infrastructural development and access to health services. Predominantly, the residents of these communities were Yorubas and were mostly farmers, traders and artisans. The population of Ife-North LGA, according to the 2006 population census was 153, 694 [31].

We used the methodology previously described by Mbada et al. [32] in this study. Hence, a fishbowl technique (i.e. a non-probability method) was used to randomly select three political or electoral wards from each of the three communities. A political or electoral ward in the Nigeria’s context is a subdivision of a LGA typically delineated as an administrative division for electoral purposes but has found other demographic usefulness. In this study, households within each political ward were considered as the Primary Sampling Unit (PSU). In order to survey these PSUs, houses were randomly selected. The first house to be surveyed was chosen by ballot because most of the houses in these rural settings were not enumerated (numbered); afterwards alternate houses were consecutively enlisted. Every consenting respondent in the enlisted houses who was 18 years older and has resided in these communities for no less than twelve months were surveyed in each of the PSU**,** until the estimated sample size was attained.

The sample size formula for populations greater than 10,000 [33] was adopted in this study, i.e. n = Z^2^pq/d^2^, where: *n* = the desired sample size (when population is greater than 10,000); *Z* = the standard normal deviate, set at 1.96 corresponding to 95% confidence level; p, the proportion of persons requiring physiotherapy services (50% i.e. 0.05) was used in line with a similar study by Igwesi-Chidobe et al. [20]; while q was 1.0-p, and d was error tolerated, set at 0.05 [20]. Thus, n = (1.962 × 0.5 × 0.5)/(0.05)^2^ = 384. Subsequently, the sample size was rounded off to 400 to accommodate for refusal to participate and invalid data.

A questionnaire containing both open- and close-ended questions developed from similar previous studies [20, 34] was used as the survey instrument in this study. The questionnaire contained three sections (see Additional file 1). Section one of the questionnaire required information on socio-demographic profile of the respondents; section two sought information on the availability and utilization of Physiotherapy services in rural setting; section three sought information on improving community Physiotherapy. Information on lifetime utilization (i.e. implies those who had received Physiotherapy in their lifetime) and point utilization (i.e. those who are currently receiving Physiotherapy services) was also obtained. The questionnaire contained largely Yes/No response options and other checklist types of questions on awareness, accessibility and utilization of physiotherapy services, as well as Likert-scaled questions on factors influencing utilization of physiotherapy services. The open-ended parts of the questionnaire were intended to elicit any other answer apart from the options already provided for the respondents to choose from. The questionnaire was subject to content validation by experts in community-based studies and the internal consistency of the questionnaire was tested in a pilot study among 20 community dwelling individuals resident in another neighboring community yielding an excellent overall internal reliability of Cronbach’s alpha of 0.88.

### Statistical analysis

Descriptive statistics of percentages, mean and standard deviation was used to summarize the data. Inferential statistics of Chi Square was used to test the association between Physiotherapy utilization and each socio-demographic variables and respondents’ belief system. Logistic regression was used to predict factors associated with Physiotherapy utilization. Statistical significance was set at *p* ≤ 0.05. Statistical Package for Social Science (SPSS) version 16.0 (SPSS Inc., Chicago, IL) was used for analysis.

## Results

A total of 336 individuals responded in this study yielding a response rate of 84.0%. The mean age of the respondents was 34.1 ± 16.2 years. The respondents were mostly within the age range of 20–30 years (45.5%). About 43.5% of them had tertiary level educational qualification (Table 1). Table 2 showed that 60.1% of the respondents were aware of Physiotherapy services via friends (36.1%), mass media (26.2%) and Doctor/Nurses referral (23.2%). The most linked media of awareness of physiotherapy was Television (56.6%) (through awareness and educational talks). Only 16.7% of respondents were referred for Physiotherapy and utilized their referrals.

**Table 1:** Socio-demographic characteristics of the respondents (N = 336)

Variable	Frequency	Percentage
Sex		
Male	166	49.4
Female	170	50.6
Age group		
< 20 years	36	10.7
20–30 years	153	45.5
31–40 years	57	17.0
41–50 years	32	9.5
51–60 years	27	8.0
> 60 years	31	9.2
Marital status		
Single	178	53.0
Married	140	41.7
Divorced	5	1.5
Separated	13	3.9
Educational status		
Primary	39	11.6
Secondary	113	33.6
Tertiary	146	43.5
Others	38	11.3
Religion		
Islam	95	28.3
Christianity	214	63.7
Traditionalism	23	6.8
Others	4	1.2
Occupation		
Farming	32	9.5
Civil service	29	8.6
Artisan	44	13.1
Business	47	14.0
Student	143	42.6
Unemployed	13	3.9
Transporter	11	3.3
Retiree	13	3.9
Others	4	1.2
Ethnicity		
Yoruba	282	83.9
Igbo	40	11.9
Hausa	10	3.0
Others	4	1.2

**Table 2:** Awareness and referral for physiotherapy services among the respondents

Variable	Frequency	Percentage
Are you aware of physiotherapy? (N = 336)		
Yes	202	60.1
No	134	39.9
Who introduced physiotherapy to you? (*n* = 202)		
Doctor	34	16.8
Midwives/Nurses	13	6.4
Relatives	29	14.3
Friend	73	36.1
Mass media	53	26.2
Media source of awareness of physiotherapy services (*n* = 53)		
Radio	7	13.2
Television	30	56.6
Advert	12	22.6
Posters	4	7.5
Referred for physiotherapy? (N = 336)		
Yes	56	16.7
No	280	83.3
Referral Source (*n* = 56)		
Doctor	34	60.7
Nurse	17	30.4
Lab. Scientist	2	3.6
Radiologist	2	3.6
Bone setter	1	1.8
Attendance to referral (n = 56)		
Yes	5	8.9
No	51	90.1

**Fig. 1:**
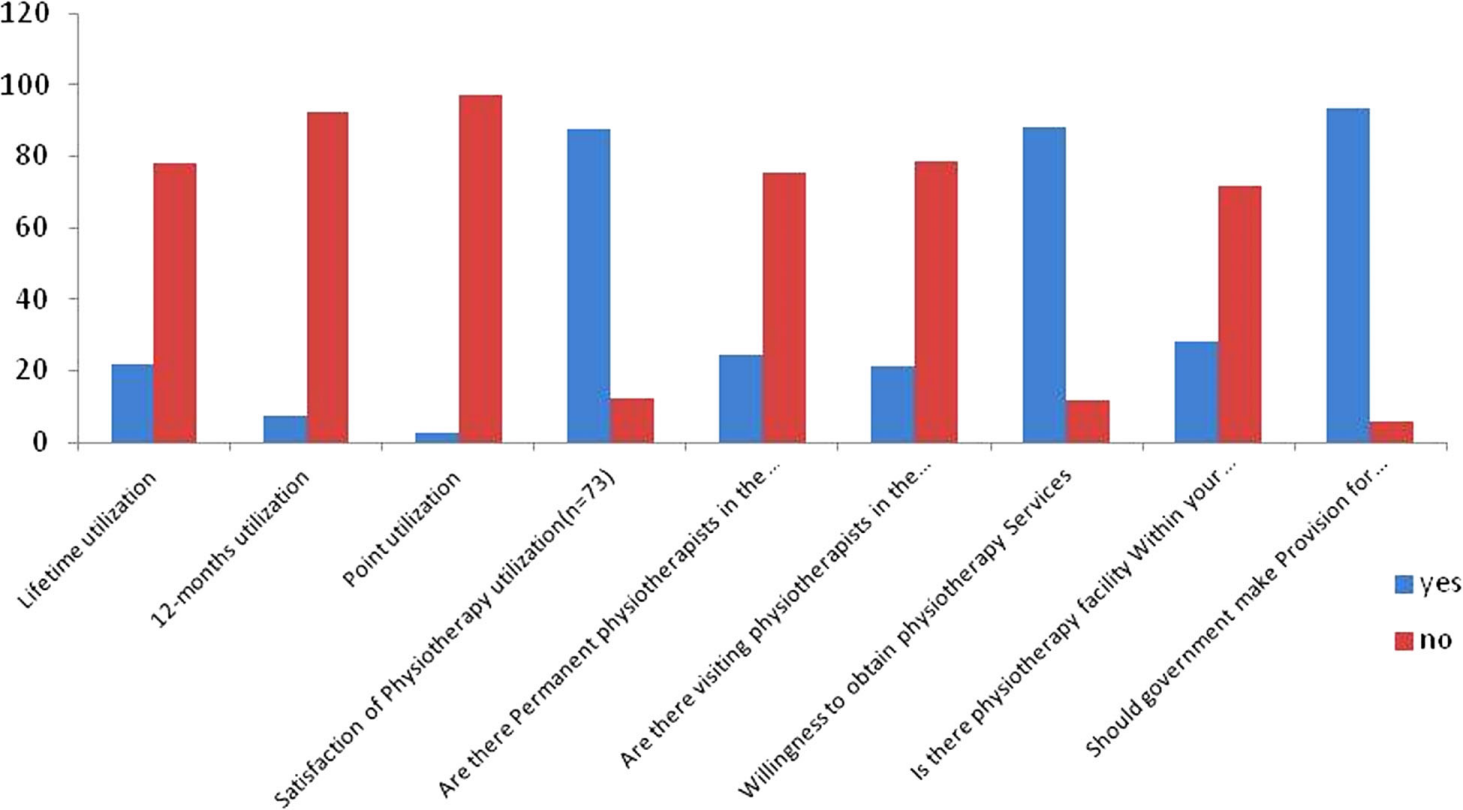
Prevalence and pattern of physiotherapy utilization among respondents in % (*N* = 336)

**Fig. 2:**
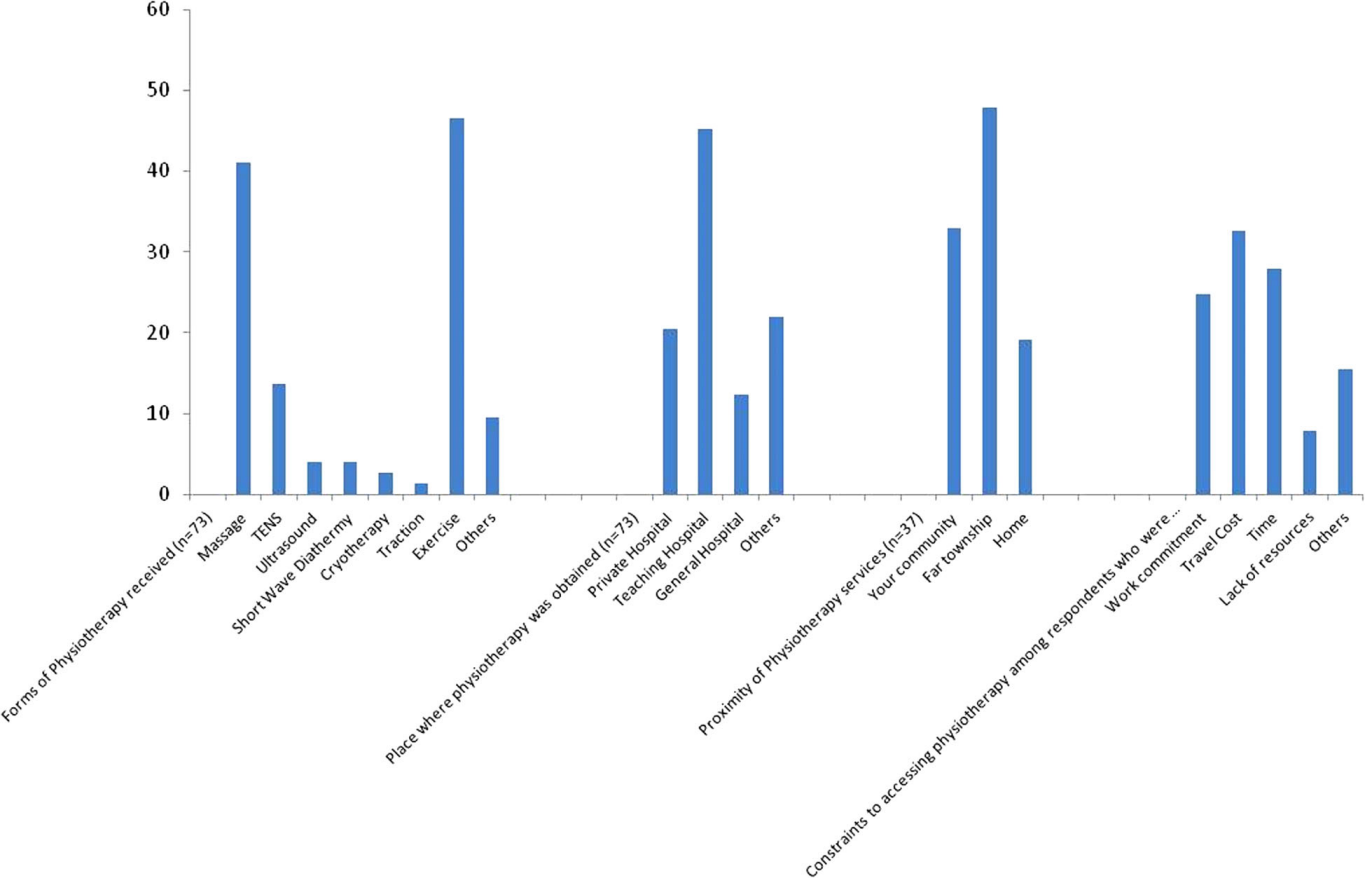
Availability and supply of physiotherapy services to respondents

Figure 1 showed the pattern of Physiotherapy utilization among the respondents. There were three categories of respondents who had utilized Physiotherapy at one time or the other. Lifetime utilization was 21.7%. 12-month utilization and point utilization of Physiotherapy among the respondent was 7.4 and 2.7% respectively. The pattern of utilization of Physiotherapy showed that 46.6% had exercise, 41.1% had soft tissue mobilization (massage), and 13.7% had one form of traction as a means of treatment (Fig. 2). Majority (87.7%) of respondents who have had Physiotherapy services reported satisfaction with treatment. A large proportion (88.3%) of respondents indicated their readiness and willingness to have Physiotherapy, if need arises. Constraints to obtaining Physiotherapy were travel cost (32.6%), time (27.9%) and work commitment (24.8%) (Fig. 2).

Results on availability and supply of Physiotherapy services to respondents are presented in Fig. 2. Physiotherapy services were mostly available in teaching hospital (45.2%) and private hospitals (20.5%). Physiotherapy services were less accessible to the communities as most of the respondents indicated that available services were at far township (47.9%). Respondents indicated that Physiotherapy services were mostly through non-permanent (24.7%) and visiting Physiotherapists (21.4%), respectively.

The association between Physiotherapy utilization, respondents’ beliefs and socio-demographic variables is presented in Table 3. There was significant association between belief on pain as “spiritual” and lifetime Physiotherapy utilization (χ^2^ = 7.826; *p* = 0.020). The respondents that disagreed about belief on pain as “spiritual” had more lifetime Physiotherapy utilization. There was significant association between Physiotherapy utilization and each of religious belief (χ^2^ = 14.181; *p* = 0.001) and stigma-related reasons (χ^2^ = 9.855; *p* = 0.007). Those who disagree that their religious belief was against physiotherapy had more Physiotherapy utilization. Also, those who disagreed that stigma attached to their condition prevented them from seeking physiotherapy had more Physiotherapy utilization. There was significant association between Physiotherapy utilization and educational status (χ^2^ = 11.980; p = 0.007). Respondents who had higher educational level reported more utilization of physiotherapy services.

**Table 3:** Association between Lifetime physiotherapy utilization and respondents’ beliefs and socio-demographic variables

Variable	Physiotherapy utilization	
	χ^2^	*P*- value
Belief		
I belief my pain is spiritual	7.826	0.020
My religious belief is against physiotherapy	14.181	0.001
Communication barrier discourages seeking physiotherapy	3.161	0.206
Difficulty in getting to where physiotherapy is available	3.249	0.197
Stigma attached to my condition prevents me from seeking Physiotherapy	9.855	0.007
Socio-demographics		
Sex	0.658	0.417
Age group	3.006	0.699
Marital status	6.049	0.109
Educational status	11.980	0.007
Religion	4.353	0.226
Occupation	15.234	0.550
Ethnicity	0.525	0.913

Out of eleven factors entered into logistic regression analysis using forward conditional method, only five factors (my religious belief is against physiotherapy, difficulty in getting to where physiotherapy is available, educational status, occupation and marital status) were paired as significant factors associated with Physiotherapy utilization (Table 4). Those who ‘agree’ and ‘not sure’ that their religious belief is against physiotherapy were 92% [Odd ratio (OR) = 0.08; Confidence interval (CI) = 0.01–0.62] and 83% (OR = 0.17; CI = 0.05–0.55) less likely to utilize physiotherapy services respectively compared with those who ‘disagree’. Those who ‘agree’ that there were difficulty getting to where physiotherapy was available were 7 times more likely to utilize physiotherapy services compared with those who ‘disagree’(OR = 6.75; CI = 1.71–26.61). The participants with primary, secondary and tertiary education were 14.3 (CI = 1.4–142.6), 13.9 (CI = 1.5–132.6) and 26 (CI = 2.6–255.6) times more likely to utilize physiotherapy services respectively compared with other forms of educational status. The single and the married were 0.173 (CI = 0.033–0.911) and 0.165 (CI = 0.039–0.706) less likely to utilize physiotherapy services compared with participants who were separated from their spouses.

**Table 4:** Logistic regression of Lifetime physiotherapy utilization and associated factors

Variable	B	SE	OR	95%CI	P
My religious belief is against physiotherapy					
Disagree (ref)					
Agree	−2.562	1.066	0.077	0.010–0.624	0.016
Not sure	−1.777	0.604	0.169	0.052–0.552	0.003
Difficulty in getting to where physiotherapy is available					
Disagree (ref)					
Agree	1.909	0.700	6.748	1.711–26.612	0.006
Not sure	−0.140	0.459	0.869	0.354–2.138	0.760
Educational status					
Others (ref)					
Primary	2.661	1.173	14.305	1.435–142.625	0.023
Secondary	2.635	1.149	13.947	1.467–132.633	0.022
Tertiary	3.259	1.166	26.019	2.648–255.649	0.005
Occupation					
Others (ref)					
Farming	−2.625	1.796	0.072	0.002–2.446	0.144
Civil service	−2.841	1.798	0.058	0.002–1.981	0.114
Artisan	−3.210	1.763	0.040	0.001–1.278	0.069
Business	−4.409	1.797	0.012	0.000–0.412	0.014
Student	−3.354	1.801	0.035	0.001–1.192	0.063
Unemployed	−2.778	1.839	0.062	0.002–2.285	0.131
Transporter	−3.481	2.055	0.031	0.001–1.728	0.090
Retiree	−2.098	1.783	0.123	0.004–4.043	0.239
Marital status					
Separated (ref)					
Single	−1.753	0.847	0.173	0.033–0.911	0.038
Married	−1.800	0.741	0.165	0.039–0.706	0.015
Divorced	−21.292	17,054.285	0.000	0.000–0.000	0.999

## Discussion

This study characterized and assessed associated factors for utilization and supply of community Physiotherapy in rural communities in Nigeria. Rate of referrals for physiotherapy among the rural residents in this study was abysmally low (16.7%). It is adducible that the low rate recorded in the present study is as a result of limited availability of Physiotherapy services and/or physicians’ apathy towards referral for Physiotherapy services that is still commonplace in the study setting. In consonance with this study’s submission, Odebiyi et al. [35] found that 57% of physicians in Nigeria do refer patients for Physiotherapy. The finding from this study reveals that the lifetime utilization of Physiotherapy was 21.7% while the 12-month and point utilization rates were 7.4 and 2.7%. While there is an apparent paucity of literature on Physiotherapy utilization, however, lifetime utilization rate observed in this study was lower than 30.2% reported in a Brazilian study [36]. Also, the 12-month utilization of Physiotherapy rate (7.4%) observed in this study was lower than those reported in studies from Netherlands (Curaçao) and Israel with prevalence rates of 8.8% [37] and 9% [38]. These differences buttress our submission that Physiotherapy was less utilized in Nigerian rural communities than most other countries, probably due to sparse availability of Physiotherapy services. Accordingly, McFadden [39] reported that if Physiotherapy services were available in rural and under-served areas, the services were likely to be used. Therefore, the foregoing has significant policy implication for health decision makers in Nigeria to pay attention to providing Physiotherapy services for the rural dwellers which could hopefully enhance its utilization.

Exercise and massage were the most utilized Physiotherapy services in the study setting. In agreement with this finding, an earlier study on Physiotherapy identity in the same study setting showed that majority of the respondents viewed a Physiotherapist as one who; teaches exercises to strengthen muscles (89.2%), teaches people how to walk (83.1%), and gives massage (81.5%) [38]. In the absence of structural facilities for Physiotherapy and permanent or resident Physiotherapists in rural communities, it is thus likely that any form of itinerant Physiotherapy services will involve manual therapy, exercise and massage than modality or equipment based treatments (such as ultrasound therapy, shortwave diathermy, and multi-gym interventions). At the same time, anecdotal observation on belief in the effectiveness of massage and other forms of joints and bone manipulations among the residents may have influenced the practices in the study communities.

Major constraints to obtaining Physiotherapy in the study setting include travel cost, time and work commitment and lack of resources/facilities for Physiotherapy. These constraints were similar to what authors of previous studies had noted [4, 16, 20, 39]. In line with the finding of this study, a predictive model confirms that individuals who agreed that they had difficulty in getting to where physiotherapy was available were seven times more likely to utilize physiotherapy service. It is conceivable from this study that those who utilized Physiotherapy services had pressing rehabilitation needs which override their present constraints or limitations in getting physiotherapy. While majority of the rural dwellers in this study who have utilized Physiotherapy services reported satisfaction with treatment, seeking utilization in the first instance, may not be unconnected to the level of awareness of these individuals. This present study showed a 60% awareness rate for Physiotherapy services. This current finding is an improvement on a previous study by Mbada et al. [32] which was conducted in the same rural setting where only 17% of the respondents were aware of Physiotherapy as a profession. It is therefore adducible that having a high proportion of young respondents in the study population has influenced the awareness level. The respondents in this study were relatively young (34.1 ± 16.2 years). Young people are believed to be conversant with trends and information even of things not readily available in their environment [40]. Furthermore, Mbada et al. [32] reported that the identity of Physiotherapy as a profession could be compromised for lack of differentiation from related health care professions. The authors also posited that this lack of differentiation could constitute an obstacle to accessing Physiotherapy services. Similar patterns of findings relating to having relatively low level of awareness about physiotherapy has been confirmed in a systematic review which concludes that physiotherapy remained a crucial partner in health care system but yet its awareness was relatively low [41].

The finding of this study further revealed that awareness of Physiotherapy in the study setting were mainly via friends (21.1%) and mass media (15.8%) than through other medical personnel. Television was the most implicated media (8.9%). These findings confirmed the relevance of human personnel and mass media in promoting awareness of information about Physiotherapy. In agreement, the Chartered Society for Physiotherapy in addressing strategic issues affecting Physiotherapy identified the media as veritable in raising the profile of Physiotherapy [42]. Although, information about Physiotherapy in Nigeria, either as advertisement or professional promotion in the media was low, however, rural community dwellers seem to always be in tune with information through the use of portable radio sets or social media using android phones. Therefore, it is conceivable that media, especially, radio and the social media may be effective in promoting the awareness of Physiotherapy in these rural communities compared with earlier reports.

From this study, availability and supply of Physiotherapy services were mostly at the township teaching hospital (47.9%) and private hospitals (20.5%). Furthermore, Physiotherapy services within the communities were mostly on ad-hoc basis (24.7%) and by visiting Physiotherapists (21.4%). These findings conveyed an apparent dearth of Physiotherapy services and absence of rehabilitative facilities in the rural communities. Gupta et al. [43] submits that rehabilitation professions human resources were mostly ignored aspect of health personnel and services for strengthening and development probably due to lack of evidence based research to inform policy strategies and advocacy. Despite global health actions at promoting and bringing health care to rural and remote communities [44], it seems that the international communities were yet to consolidate guidelines for community-based rehabilitation [44]. Human resources for rehabilitation were often absent from national health sector plans and reviews or human resource for health development strategies [45]. Evaluating the rehabilitation health workers’ accessibility is a vital starting point for understanding the ability of health systems to meet health-related rehabilitation service goals in a nation. Rehabilitation workforce profiling have been reported by a few studies and estimate gaps with diverse data sourcing limited to a definite nation/region and focusing on single profession or practice modality [46–49]. However, there was an apparent dearth of such studies in sub-Sahara Africa. Corroborating this submission, Haig et al. [50] facetiously concluded, taking into account the lack of documentation on physical and rehabilitation medicine in sub-Saharan Africa, the chance of a person with a disability in sub-Saharan Africa meeting a physician with specialist skills was limited. Thus, research on determinants of rehabilitation supply is warranted with a view on its importance on policy and for pragmatic actions.

From this study, a majority of the respondents indicated their readiness and willingness to have Physiotherapy services, if the need arise. However, Physiotherapy utilization was found to be significantly influenced by each of belief of pain as “spiritual”, religious belief, stigma-related reasons and educational status. Of the socio-economic status indicators, education was found to be a significant indicator of Physiotherapy utilization. This above stated finding was further buttressed by the results of the regression analysis that level of education determined physiotherapy utilization significantly. Specifically, this study revealed that respondents with tertiary education status (28.8%) utilized Physiotherapy. This rate was higher than those observed among respondents with lower educational status. Demographic characteristics have been implicated in many studies to significantly influence health seeking behavior [51, 52]. Furthermore, traditional beliefs about pain and religious inclination significantly influence uptake of Physiotherapy services in this study. Patients’ beliefs, fear of pain, pain behaviors and response to pain were bio-behavioural factors affecting pain and disability [53]. Patients’ beliefs about the cause of their pain and anticipated effects of treatments may also influence whether they take up a particular treatment and the likely outcome of treatment [54]. This was supported by our results that those who have negative beliefs were 92% less likely to utilize physiotherapy services. The traditional method of Physiotherapy assessment, intervention and treatment using different modalities of treatment often proves ineffective with patients that have biased mind, negative beliefs, wrong knowledge and negative practices with regards to low back pain [53].

The findings of this study might help policy makers to put in place appropriate policy to organize the rehabilitation health services in terms of its utilization and supply. Improvements in availability and access to community-based rehabilitation were warranted, and should increase Physiotherapy utilization in rural communities in Nigeria. Physiotherapy should be a statutory component in primary care, and there should be a dedicated custom-built Physiotherapy clinic in any public facility especially in rural settings.

### Limitations

Caution should be taken in interpreting this study results. The sample of convenience used to enlist or select three rural communities, and the relatively small sample size may limit the generalizability of the findings of this study to the whole rural communities in Nigeria, as well as extrapolating the findings to other settings. Furthermore, as in many cross-sectional studies, causal relationship between associated variables cannot be assumed in this study.

## Conclusion

Utilization and supply of Physiotherapy services in Nigeria rural community was low. Low utilization of Physiotherapy services in Nigeria rural communities were most significantly influenced by low educational status and beliefs about pain. Understanding the determinants of Physiotherapy utilization may facilitate efficient resource allocation to provide services to address the needs of rural communities, and improve health outcomes of patients.

## Additional file

Additional file 1: Title of data: Survey questionnaire. The tool used to assess the supply and utilization of physiotherapy services. (DOCX 87 kb)

## Abbreviations

CI: Confidence interval
LGA: Local Government Area
OR: Odd ratio
PSU: Primary Sampling Unit
SPSS: Statistical Package for Social Science
WHO: World Health Organization
