# Supplementary material for: Characteristics and determinants of community physiotherapy utilization and supply
Source: BMC Health Serv Res. 2019 Mar 14;19:168. doi: 10.1186/s12913-019-3994-4 (PMC6419371; doi:10.1186/s12913-019-3994-4)
Supplement: Supplementary file 1 — Title of data: Survey questionnaire. The tool used to assess the supply and utilization of physiotherapy services. (DOCX 87 kb) [file 12913_2019_3994_MOESM1_ESM.docx]

**DEPARTMENT OF MEDICAL REHABILITATION**

**COLLEGE OF HEALTH SCIENCES**

**OBAFEMI AWOLOWO UNIVERSITY**

**ILE-IFE, NIGERIA**

**SURVEY QUESTIONNAIRE**

The purpose of this study is to determine the supply and utilization of physiotherapy services in your locality. The information obtained from this study may guide in policy-making on availability and accessibility of physiotherapy services in rural settings. You are please requested to answer the questions as honestly as you can. The information obtained in this study will be kept strictly confidential and will be for academic purpose only. You are free to withdraw from this study at any time without sentiment. Thank you for making out time to fill this questionnaire.

**SECTION A: Socio-demographic data**

1. Age:___________

2. Sex: Male Female

3. Marital status: Single Married Divorced Separated

4. Religion: Islam Christianity Traditionalism

Others (please state)__________________________________

5. Highest educational qualification: Primary Secondary Tertiary

Others (please state)___________________

6. Ethnicity: Yoruba Igbo Hausa

Others (please state)________________

7. Occupation:

Farmer Civil Servant Artisan Business/Trader

Student Unemployed Driver/Rider Retiree

Others (please state)____________________________

**SECTION B – Information on availability and utilization of physiotherapy services**

1. Are you aware of Physiotherapy? Yes No

**2**. Have you ever received any form of physiotherapy? Yes No

**3**. If you answered “Yes” to Question 2, were you satisfied with the services? Yes No

**4**. Did you receive any physiotherapy service in the last 12 months? Yes No

**5**. Are you presently on any form of physiotherapy service? Yes No

**6**. How did you get to know about physiotherapy services? Through a

Doctor Midwife/Nurse Relative Friend

Mass Media

**7**. If through media, what type?

Radio Television Advert Posters

**8**. Have you ever been referred for physiotherapy before? Yes No

**9**. Who referred you?

Medical Doctor Nurse Medical Laboratory Scientist

Radiologist Massager Bone Setter

Others…………………………………………………………….

**10**. Did you go for the referral? Yes No

**11**. What form(s) of physiotherapy did you receive? (Please check all that apply) -

Massage therapy TENS Ultrasound

Shortwave Diathermy Cryotherapy Traction

Exercise Others (please state)_____________________________

**12**. Where did you receive physiotherapy services?

Private Hospital Teaching Hospital General Hospital

Others:__________________________________________

**13.** Was the physiotherapy services rendered in:

(a) your community (b) a far township from (c) your home

**14.** Are there permanent physiotherapists working in your community? Yes No

**15.** Is there visiting physiotherapist in your community? Yes No

**16**. If you answered “No” to the Question 2 “Have you ever received any form of physiotherapy?”, why? Work commitment Travel cost

Time Lack of centre resources

Others­­­­­­______________________________________________________

**17**. Rate your level of agreement with the statement below on utilization of Physiotherapy service

| Questions | Strongly Agreed | Agreed | Neutral | Disagreed | Strongly Disagreed |
| --- | --- | --- | --- | --- | --- |
| My belief about pain and disorders as spiritual affects my utilization of physiotherapy |  |  |  |  |  |
| My religious belief does not permit me to seek medical attention for my condition |  |  |  |  |  |
| Communication barrier that I may have with physiotherapist made me not to attend physiotherapy |  |  |  |  |  |
| I did not allow attend physiotherapy because getting to the clinic is difficult |  |  |  |  |  |
| Because of the stigma attached to my condition, I did not attend physiotherapy |  |  |  |  |  |

**SECTION C: Information on improving community physiotherapy**

1. Will you be willing to obtain physiotherapy services? Yes No
2. Is there any physiotherapy facility around your environment? Yes No
3. If “Yes” to Question 2, is it government owned? Yes No
4. Will you be willing to attend a physiotherapy facility closer to you? Yes No
5. Should government make provision for physiotherapy facilities around your environment? Yes No

**For enquiry contact**

**Dr. Chidozie E. Mbada**

**E-mail:** [**doziembada@yahoo.com**](mailto:doziembada@yahoo.com)

**Thank you**
